# Microbial biotechnology in the effort to end hunger

**DOI:** 10.1111/1751-7915.14295

**Published:** 2023-06-23

**Authors:** Juan Luis Ramos, Patricia Bernal, Davinia Salvachúa

**Affiliations:** ^1^ Consejo Superior de Investigaciones Científicas – Estación Experimental del Zaidin Granada Spain; ^2^ Departamento de Microbiología, Facultad de Biología Universidad de Sevilla Sevilla Spain; ^3^ Renewable Resources and Enabling Sciences Center National Renewable Energy Laboratory Golden Colorado USA

## SETTING THE SCENE

Hunger remains one of the most pressing challenges of our time. The global population is expected to reach nearly 10 billion by 2050. As populations continue to rise, hunger will also increase; this is partly because we are losing arable land for crop production every year. Emerging pathogens and climate change are also affecting global food production and security, intensifying the challenges associated with meeting the growing demands of the population (Batista & Singh, 2021; Buchholz et al., 2018; Sessitsch et al., 2018; Thomashow et al., 2019).

Hunger and malnutrition can be further aggravated by food distribution chains that do not align with population needs and are unaware of this global crisis. Food waste also contributes to this issue, with an estimated one third of food being discarded or wasted (Buchholz et al., 2018). Despite efforts to quell these losses, the percentage of wasted food has remained constant over the past 40 years. For instance, a Swiss study, which focused on the potato industry, revealed that over 55% of the initial fresh potato harvest and 40% of processed potatoes are lost due to various factors such as pathogen infections, water loss and premature sprouting during storage (Paliwal et al., 2022; Willersinn et al., 2015). The post‐harvest microbiota of crops and its impact on storage stability is an area that warrants further research, holding the potential to mitigate crop losses (Buchholz et al., 2018).

The UN has set a series of goals with the aim of fostering sustainability on our planet. Among these goals, Sustainable Development Goal (SDG) 2 aims to END HUNGER by 2030. However, under the current global conditions, this will be difficult to achieve without significant efforts by governments and other stakeholders. It is imperative that we prioritize the increase of agricultural production while simultaneously exploring sustainable resources for crop production (Sessitsch et al., 2018). Technologies such as precision agriculture, genetic engineering of crops and the use of bioinoculants and biopesticides hold tremendous potential to enhance crop production.

In addition to the aforementioned priorities, agriculture in the twenty‐first century also faces the challenge of reducing our reliance on inorganic fertilizers. While synthetic fertilizers have significantly increased global crop production and reduced poverty and hunger, they have also caused massive and widespread pollution, biodiversity loss and land degradation (Matassa et al., 2022). Rationally designed fertilizers can mitigate losses and improve fertilizer efficiency. However, their performance must be demonstrated to be consistent across different soil types and climates. Innovative and environmentally friendly solutions are crucial to ensure sufficient and sustainable agricultural production while minimizing environmental impact.

## THE SOIL CRISIS

Healthy soil is vital for sustainable agriculture as it supplies essential nutrients to plants and contains soil microbes that play a critical role in nutrient bioavailability. The soil microbial community is also essential for protecting plants from biotic and abiotic stress, which ultimately boosts plant health and productivity. The plant microbiome, or the microbial community associated to all plant organs and tissues, is considered a functional extension of plants. This connectivity lends weight to the adage, ‘healthy soils, healthy roots, healthy plants, healthy people’.

Anthropogenic activities, extreme weather events and global warming are causing unprecedented soil deterioration, desertification and erosive loss. This has led to the current soil crisis, compromising the provision of essential ecosystem services, such as biogeochemical cycling, waste recycling and water purification. Most of these services are mediated by an array of microbial‐driven reactions. Timmis and Ramos (2021) proposed the concept of treating soils as patients in need of healthcare to address these insults and tackle the soil crisis. They proposed the creation of a soil healthcare system with treatments that are informed by evidence‐based data and which can be integrated into policies for land use, conservation, restoration, prophylactic measures, monitoring and crisis response. The implementation of such systems needs to be coordinated globally to slow and ultimately reverse soil loss. Securing the health of soils and waters is a critical challenge in the face of climate change, and is essential to achieving the United Nations' SDGs.

In the wake of the Green Revolution, our soils have suffered a massive influx of nitrogen and phosphorus from chemical fertilizers (Matassa et al., 2022; De Zutter et al., 2021). This imbalance has endangered environmental health and sustainability. The use of nitrogen and phosphorous fertilizers to increase crop productivity has led to negative long‐term effects on plant–soil‐microbe networks, microbial metabolism and functionality. A 150‐year monitoring study at Rothampsted Experimental Station (UK) has shown that inorganic nitrogen reduces rhizosphere dependence on root‐derived carbon and weakens plant–microbe networks (Huang et al., 2019).

Another key requirement for sustainable agriculture is the reduction of direct and indirect greenhouse gas emissions, which are strongly linked to fertilizer usage. Moving towards the use of organic fertilizers, that is, manure, with slower nutrient release patterns, which depend on microbial activities, is essential (Shi et al., 2023; Thomas et al., 2020). The shift to microbially released phosphate is particularly important, given the eutrophic effects of phosphate chemical fertilizer runoff and declining phosphorous stocks worldwide.

## PLANT–MICROBE INTERACTIONS TO ENHANCE PLANT PRODUCTIVITY

Plants rely on soil microbes within their rhizosphere for growth and development. Referred to as plant growth promoting microbes (PGPM), these vital soil components, which include bacteria and fungi, improve plant yield and function as biofertilizers. PGPM promote growth through processes such as nitrogen fixation, phosphate solubilization, organic nitrogen mineralization, removal of pollutants, biocontrol, methane emission mitigation and carbon sequestration (Jing et al., 2020; Lindström & Mousavi, 2020; Sakarika et al., 2020; Wang et al., 2021). PGPM also contribute to and modulate plant hormone levels, thereby inducing systemic resistance in plants (Sakarika et al., 2020). There is a growing trend towards developing microbial consortia for use as bioinoculants, as these can be more effective than single microorganisms. The simplest consortium is the combination of two or more independently isolated PGPM; however, current research is focused on developing synthetic communities (SynComs) (Hu et al., 2022). These take into account the diverse plant–microbe chemical and molecular communication networks at play. The development of SynComs is informed using multi‐omics data, which enables the characterization of the taxonomy and beneficial properties of core microbial communities that colonize different plant compartments (Sakarika et al., 2020). The protocols being used also facilitate the selection of microbes in situ—in direct association with plants—thereby ensuring the adequate performance of these microbes in real‐life scenarios. Despite the remaining challenges, this approach is shedding light on the biochemical and genetic mechanisms that govern the interplay between plants and endophytes—insights that are key to unlocking the biotic repertoire necessary for sustainable plant management (Lindström & Mousavi, 2020; Sakarika et al., 2020).

Disease‐suppressive soils, which can control soil‐borne pathogens, rely on the delicate balance between diverse plant‐associated microbial communities (Bakker & Berendsen, 2022). The microbiome is influenced by both the pathogen and the plant, making it challenging to distinguish signals originating from each. Moreover, aboveground plant parts are attacked by pathogens and pests, which can impact the root‐associated microbiome. Insect infestations have been shown to recruit fluorescent pseudomonads with insect‐killing capacity, and such interactions can lead to significant effects on the root microbiome (Paliwal et al., 2022). Recent studies have revealed that biofertilizers can reshape the rhizosphere bacterial community, enhancing beneficial microbial consortia capable of suppressing plant diseases, such as banana Panama disease (Shen et al., 2021). Thus, understanding the soil microbiome is a key to raising defences against invading plant pathogens.

Biofertilizers and biofungicides are successful examples of microbiome‐based technologies that rely on inoculation with beneficial microbes (Lindström & Mousavi, 2020; Thomashow et al., 2019). A benefit of this approach is that microbes can be cultivated in all geographical areas. They can also be customized, based on soil and climatic conditions, to maximize beneficial outcomes. To meet Sustainable Development Goals, we need to explore, combine and optimize various strategies while ensuring their scalability and adaptability to diverse settings and conditions (Hu et al., 2022). Although significant work lies ahead, forging this path will lead to new high‐quality rural jobs and will foster healthier soils, plants and people.

## FUNDING INFORMATION

Comisión Interministerial de Ciencia y Tecnología, (Grant/Award Number: 'PID20211234690BI00').
